# Increased Avian Diversity Is Associated with Lower Incidence of Human West Nile Infection: Observation of the Dilution Effect

**DOI:** 10.1371/journal.pone.0002488

**Published:** 2008-06-25

**Authors:** John P. Swaddle, Stavros E. Calos

**Affiliations:** 1 National Center for Ecological Analysis and Synthesis, University of California Santa Barbara, Santa Barbara, California, United States of America; 2 Institute of Integrative Bird Behavior Studies, Biology Department, College of William and Mary, Williamsburg, Virginia, United States of America; Oxford University, United Kingdom

## Abstract

Recent infectious disease models illustrate a suite of mechanisms that can result in lower incidence of disease in areas of higher disease host diversity–the ‘dilution effect’. These models are particularly applicable to human zoonoses, which are infectious diseases of wildlife that spill over into human populations. As many recent emerging infectious diseases are zoonoses, the mechanisms that underlie the ‘dilution effect’ are potentially widely applicable and could contribute greatly to our understanding of a suite of diseases. The dilution effect has largely been observed in the context of Lyme disease and the predictions of the underlying models have rarely been examined for other infectious diseases on a broad geographic scale. Here, we explored whether the dilution effect can be observed in the relationship between the incidence of human West Nile virus (WNV) infection and bird (host) diversity in the eastern US. We constructed a novel geospatial contrasts analysis that compares the small differences in avian diversity of neighboring US counties (where one county reported human cases of WNV and the other reported no cases) with associated between-county differences in human disease. We also controlled for confounding factors of climate, regional variation in mosquito vector type, urbanization, and human socioeconomic factors that are all likely to affect human disease incidence. We found there is lower incidence of human WNV in eastern US counties that have greater avian (viral host) diversity. This pattern exists when examining diversity-disease relationships both before WNV reached the US (in 1998) and once the epidemic was underway (in 2002). The robust disease-diversity relationships confirm that the dilution effect can be observed in another emerging infectious disease and illustrate an important ecosystem service provided by biodiversity, further supporting the growing view that protecting biodiversity should be considered in public health and safety plans.

## Introduction

The dilution effect is an outcome of a potentially broadly applicable set of mathematical models that could help explain human risks of contracting vector-borne zoonoses, i.e., infectious diseases that are spread among host animals by a vector and threaten to spill over into the human population [1]–[9]. Examples of zoonoses include avian influenza, anthrax, bubonic plague, Lyme disease, and West Nile virus, to name but a few. The fundamental principle underlying the dilution effect is that increased host diversity can dilute disease incidence through multiple mechanisms [1]. Such mechanisms include, in situations of increasing host diversity, a reduction in the probability of transmission of the disease from infected hosts to vectors (*transmission reduction*) [10], a reduction in the rate of encounters between hosts and infected vectors (*encounter reduction*), a reduction in the number of susceptible hosts (*susceptible host regulation*), a reduction in infected vector density (*vector regulation*), and a faster disease recovery rate among infected hosts (*recovery augmentation*) [1]. All of these mechanisms have a complementary augmentation (e.g., encounter augmentation) or reduction (i.e., recovery reduction) mechanism that can give rise to a positive relation between diversity and disease incidence in certain situations.

The mechanisms of these models and much of the empirical demonstration of effects have been explored in the context of Lyme disease, where there is lower probability of being bitten by an infected tick (vector) in woodland areas of relatively increased small mammal (host) diversity [5], [6]. However, there is a need to explore whether the dilution effect is applicable to other diseases, whether such diseases vary in incidence and risk with diversity, and to attempt to identify the mechanisms by which the dilution effect operates [1]. Meeting these demands can help inform and refocus more effective public health, conservation, and bioterrorism-preparedness strategies by identifying ways in which wildlife (host) community structure can be utilized to minimize the health, ecological, and economic consequences of emerging infectious diseases, whether these diseases emerge naturally or are introduced deliberately.

Here, we explore whether the general pattern predicted by these models (i.e., a negative relationship between disease and host diversity) occurs for a recent human zoonosis in the US, West Nile virus (WNV). Explicitly, we explore the relationships of measures of avian host community diversity and the abundance of particular avian families implicated in the spread of WNV with the incidence of the human disease in eastern US counties. We also attempt to distinguish the relative merits of some of the mechanisms that may drive a diversity-disease relationship.

West Nile virus (WNV) affects avian and mammalian populations worldwide and has become the focus of considerable conservation, veterinary, and human health concerns [11]–[15]. The virus primarily replicates within birds and is predominately spread between hosts by mosquito vectors [4], [11], [16], [17]. As the frequency of infected birds increases in local populations, the chances of incidental hosts, such as humans, being bitten and infected by a mosquito carrying WNV also increases [4].

To observe the predicted dilution effect in the context of WNV transmission the vector (mosquito) must feed from numerous host (bird) species and these hosts must vary substantially in their competence as disease hosts (i.e., the product of species susceptibility, infectiousness, and the duration of the infection, as defined in [16]) and the least competent hosts must increase in relative abundance in more diverse host communities; and/or transmission of the virus within a host species must be greater than transmission among host species. These conditions appear to be met for WNV transmission: the mosquitoes that spread WNV feed from multiple bird species [4], [10], [11], [15], [16] and will bite incidental hosts such as humans and horses; bird species vary substantially in their host competence [16], [18]; and some of the most competent hosts (e.g., crows, jays, finches, sparrows, and thrushes) are frequently present in low diversity (suburbanized and urbanized) avian communities, whereas the least competent hosts (e.g. coots, quail, pheasants, geese, woodpeckers, and parakeets) tend to appear in more diverse assemblages [19], [20]. The assumption that disease transmission is more likely within than among host species is less well-supported, partly because this is difficult to investigate directly in field studies. Transmission within-species by contact has been observed in the laboratory [16], [17]. This could mean that species living at high density can spread the virus to conspecifics through additional non-vector mechanisms. In terms of more likely vector transmission routes, the most prevalent vector species in the eastern US (*Culex pipens*) has notable affinities to feed from particular host species [21], thereby increasing the probability of within versus among host species transfer of the virus.

For WNV transmission, the models leading to a dilution effect predict that as the species diversity of local avian populations increases, the relative abundance of less competent avian host species will increase and/or transmission of WNV among hosts will be reduced by introducing more species. Either, or both, of these patterns will result in a lower probability of uninfected organisms contracting WNV and a lower probability of incidental hosts, such as humans, contracting WNV [1], [4]. There is preliminary evidence to indicate that increased non-passerine (non-songbird) species richness is associated with lower incidence of human WNV in Louisiana, US [4]. However, this previous study [4] compared diversity to disease after WNV could have affected avian community diversity, therefore obscuring any causal implications between avian host community structure and the risks to humans from WNV. For example, the disease could have caused a reduction in avian diversity while also infecting humans. This study also ignored information from counties that reported no human WNV cases, which may be the most informative from a public health point of view, and also did not to account for any covariation with climate variables.

Here, we report a much broader geographic examination of whether avian diversity and community structure can predict the occurrence of WNV in humans by examining associations of human WNV cases with avian community structure in the eastern US from the year before WNV was introduced to the US, 1998, [14] to the first year of the human epidemic in these states, 2002. Specifically, we related the per capita incidence of human WNV with metrics of bird diversity and the relative abundance of particular avian families, and one species, that are predicted to relate to human risks of contracting WNV: Corvidae, crows and jays [16], [22], [23]; Fringillidae, finches [24]; Passeridae, exclusively the house sparrow *Passer domesticus* in these counties [25]; Turdidae, thrushes; and the American robin *Turdus migratorius* [10], [21]. Within these analyses we attempted to control for geospatial relations in the data, regional climate variation, regional variation in vector type, human socioeconomic factors, and county urbanization, all of which may confound any relationships between avian community structure and human incidence of WNV. We predicted that there would be lower incidence of human WNV in counties with greater avian diversity and a lower relative abundance of corvids, finches, house sparrows, thrushes, and American robins.

In an attempt to assess the relative merits of the non-mutually exclusive mechanisms that could underlie a relationship between diversity and disease, we generated predictions of how aspects of diversity and community structure should relate to human WNV incidence under the principle mechanisms (Table 1). These predictions were not straightforward to form as our data are correlational, hence it is difficult to assign any particular causal mechanism to the patterns in our data. We want readers to interpret our data with this caveat in mind. However, our analyses are an important first step toward indicating how a dilution effect may be manifest for a vector-borne zoonosis across a broad geographic scale and may also help to stimulate more specific hypotheses for how each mechanism can be diagnosed in future studies. Our predictions were as follows.

**Table 1:** Mechanisms that can give rise to a negative relationship between avian diversity and human WNV incidence, with associated predictions and findings from this study.

Mechanism	Definition	Predicted pattern	Findings from this study
Transmission reduction	Reduction in the probability of transmission of WNV from infected birds to mosquitoes	Avian community evenness should be a better predictor than species richness of human WNV incidence	Avian species richness is a better predictor than community evenness of human WNV incidence
Encounter reduction	Reduction in the rate of encounters between hosts and infected mosquitoes	Avian community evenness should be a better predictor than species richness of human WNV incidence	Avian species richness is a better predictor than community evenness of human WNV incidence
Susceptible host regulation	Reduction in the number of susceptible hosts	Absolute rather than relative abundances of high-competence disease hosts should be better predictors of human WNV. Also, absolute abundance of all avian host species combined should be a positive indicator of human WNV.	Absolute abundance metrics were not better predictors of human WNV than relative abundance metrics. However, absolute abundance of all avian species combined was a good predictor of future human infection.
Vector regulation	Reduction in density of infected mosquitoes	We adjusted analyses for an estimate of vector density (i.e., an urbanization metric)	We can rule out this mechanism as vector density was accounted for in all analyses
Recovery augmentation	Faster disease recovery rate among infected hosts	Cannot be examined by this study	N/A

Transmission reduction is regulated by the relative densities of higher- versus lower-competence hosts in the local community [1]. If relatively more lower-competence or uninfected hosts are introduced into a population then the encounter rate between the vector and the more-competent infected hosts will be reduced. As community evenness captures information about the relative abundances of hosts and this information is ignored by a species richness metric, we predicted that the importance of transmission reduction will be illustrated by a relatively stronger negative relationship between disease prevalence and evenness than the relationship between disease and species richness. Encounter reduction should follow a similar pattern, as adding relatively more individuals of an additional species will reduce rates of contact that could aid transmission of the pathogen. Hence, under encounter reduction, we also expect avian community evenness to be a stronger predictor than species richness of human WNV.

Susceptible host regulation is sensitive to the absolute number of different avian host species in the community [1]. Therefore we predicted that absolute abundances of susceptible avian groups (i.e., corvids, thrushes, finches, Old World sparrows, and the American robin) should be better predictors of human WNV than relative abundances of the same groups. Additionally, the total abundance of all host species should be a predictor of human infection if this form of density-dependent mechanism operates in the transmission of WNV [1]. We excluded vector regulation from explaining patterns in our data as this mechanism relies on variation in vector density and we adjusted all of our analyses for a metric of mosquito abundance (i.e., an index of urbanization). Hence, we do not consider this mechanism any further. Investigating whether recovery augmentation can explain a diversity-disease relationship requires information about disease recovery rates in birds. We do not have such data and, therefore, cannot evaluate this particular mechanism. All of these predictions are summarized in table 1.

We found evidence for the predicted negative relationship between avian community diversity and human disease incidence, with species richness being a stronger predictor of disease than community evenness. However, many of the family-level analyses generated unexpected patterns.

## Materials and Methods

**Figure 1:**
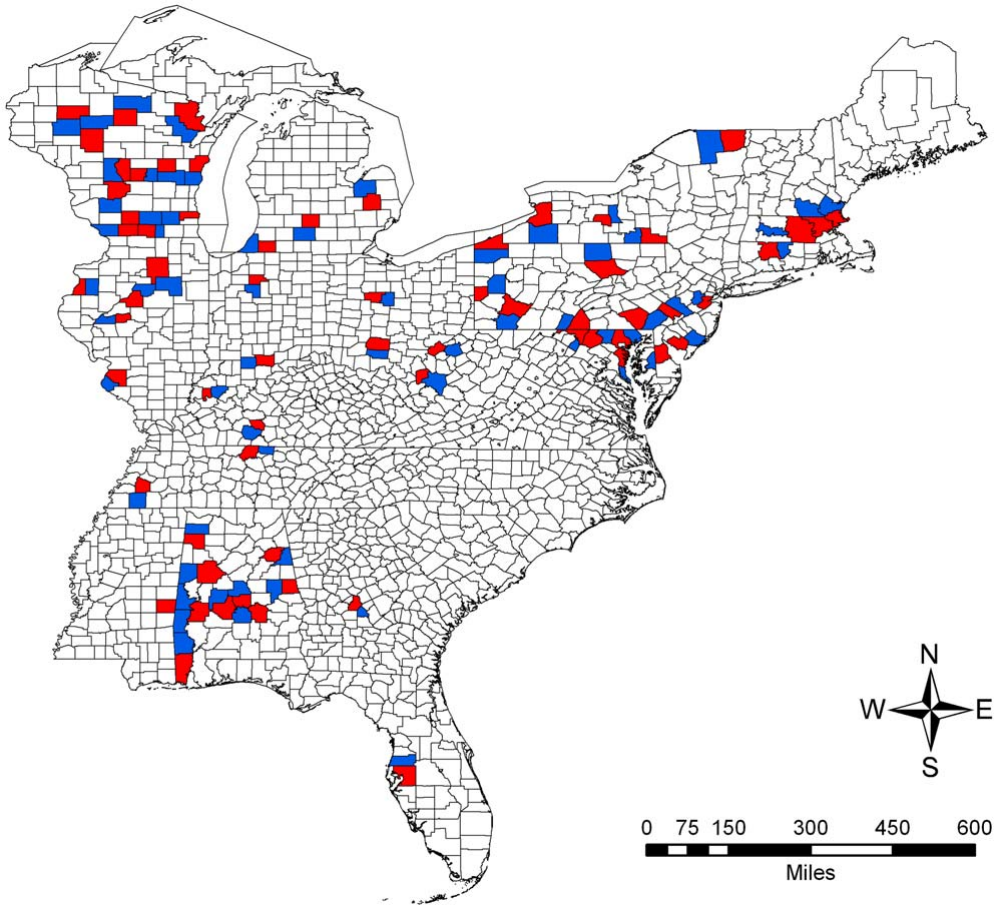
Eastern US counties used in the geospatial contrasts analyses. Red shading indicates counties that reported positive tests for human WNV in 2002; blue shading indicates counties that reported no positive cases of human WNV.

Disease prevalence can be affected by multiple factors other than host diversity. To minimize confounding factors we constructed a novel geospatial contrasts method that related avian community structure to human WNV incidence (per capita) using the between-county differences in avian community structure metrics and human disease incidence of neighboring counties in which one county did not report human WNV and the other did (Fig. 1). All counties reported avian WNV. These pairs of contrasts let us compare the disease-community structure relationships while accounting for geospatial non-independence of human disease incidence and of avian community structure, and regional variation in climate and local mosquito vector type.

We identified all neighboring pairs of counties east of the Mississippi river (i.e., the eastern US) where we could obtain human WNV and Breeding Bird Survey (BBS) data in both 1998 (before WNV was detected in the US) and in 2002 (the first year of the human epidemic in the US), and where one county reported no human infection in 2002 and the neighbor reported at least one human case. Sixty five pairs of counties met these criteria. When there was a choice of which neighbor to assign to a particular county, we selected the closest neighbor based on the distance between county centroids. Each county was used once only in the analyses and could not be counted as a neighbor for more than one other county. For each county we calculated indices of avian community structure from BBS route data, which are freely available from the US Geological Survey [26]. Specifically, we broke BBS routes into five equal sections and aligned routes to county borders. We assigned BBS data to a county if more than half of a survey route occurred in a county. We also matched the number of BBS route stops within the pairs of counties as increased sampling effort in one of a pair would likely lead to an artificially high estimate of avian diversity or relative abundance of rare species. From these BBS survey data we constructed indices of species richness and Shannon-Weiner evenness of non-passerines, passerines, and all species combined, the total number of birds sampled on a route, the relative and absolute abundance of four avian families known to be particularly competent viral hosts: Corvidae, crows and jays [16], [22], [23]; Fringillidae, finches [24]; Passeridae, Old World sparrows [15], [16], [21], [25], [27]; Turdidae, thrushes and robins; and the relative and absolute abundance of one species recently implicated in human epidemics of WNV, the American robin *Turdus migratorius* [10], [21], [28]. For a given year, the BBS survey routes are generally sampled a month before most reports of human WNV; hence the BBS data give a good representation of avian community structure during the period when humans are likely being infected. Avian and human WNV infection information was also obtained from the freely available database that is maintained by the USGS and based on annual reports issued by the Centers for Disease Control.

As one of each pair reported zero human cases, we used the incidence of the infected counties as the contrasts (i.e., difference) in human WNV incidence between the neighboring pairs. The community structure contrasts were calculated by subtracting the relevant metric scores for the infected counties from the same metric scores from their uninfected neighbors. Hence, if the uninfected counties had greater avian diversity, we would expect to see a negative relationship between the contrasts for human WNV incidence and each metric of avian diversity. Therefore, this technique rendered 65 contrasts representing the 65 pairs of counties for which there were sufficient data. These contrasts are useful for several reasons. First, they account for the non-independence of neighboring data points. Essentially, they allow us to probe, within a county pair, whether an increase in diversity from one county to another is associated with an increase or decrease in the incidence of human disease. Also, the contrasts report a difference (or change) in community structure metrics over space which can be useful in making recommendations for management strategies. For example, the contrast can be used to plan for an increase (or decrease) in a metric of community structure over a specified time interval or spatial scale and that difference may be associated with a subsequent change in human disease incidence.

We performed a principal components analysis (employing the correlation matrix method) of 2000 Census Bureau data for all counties east of the Mississippi river. The PCA generated two components with eigenvalues greater than one. The first component (human demographic PC1) explained 46.7% of the variation and loaded highly negatively with median household income and loaded positively with percentage of the population under the poverty line and the percentage of the county population that was unemployed (Table 2). We interpreted PC1 to represent an index of low socioeconomic status. The second component (human demographic PC2) explained a further 20.6% of the variation in the data and loaded highly positively with population density and negatively with the percentage of people under 5 or over 65 years of age (Table 1). Hence, we interpreted PC2 as a positive index of county urbanization. Human socioeconomic status may affect WNV incidence as lower socioeconomic populations are likely to be more susceptible to disease in general. Urbanization may affect the disease-community structure relationships as the primary mosquito vectors, *Culex spp.*, are often urban associated [21], [29], [30] and humans at higher density in urban areas may experience a higher per capita probability of contracting infectious diseases.

**Table 2:** Component matrix from the principal components analysis of original 2000 US Census Bureau data.

Variable	PC1	PC2
% of population under 5 or over 65 years of age	0.383	−0.455
Population density per square mile	−0.093	0.870
Median household income	−0.918	0.008
% of population under the poverty line	0.887	0.242
% of population that are unemployed	0.742	0.065

We performed Pearson partial correlation analyses of the spatial contrasts of human incidence of WNV in 2002 on contrasts for avian community structure in 1998 (before WNV was reported in the US) and in 2002 (the first year of the epidemic), while accounting for variation in county socioeconomic status (human demographic PC1) and urbanization (PC2). By comparing diversity-disease relationships with avian community data from before WNV was reported in the US (1998) to the time of the epidemic (2002) by ANCOVA, we explored whether the disease-community structure relationships changed as the bird populations changed in association with the WNV epidemic. Explicitly we used “year” as factor and the relevant diversity metric and the two human demographic PCs as covariates and interpreted the year-by-diversity interaction term to look for a change in the diversity-disease relationship across years.

We also performed a linear regression model selection procedure in which we started with a maximal model that had the following predictors of the contrast for human incidence of WNV: spatial contrasts for total species richness, total community evenness, nonpasserine community evenness, passerine community evenness, relative abundance of passerines to nonpasserines, relative and absolute abundances of Corvidae, Passeridae, Fringillidae, Turdidae, and American robins, the total abundance of all birds sampled on a route, and human demographic PC1 (socioeconomic status) and PC2 (urbanization). As we wanted to account for human socioeconomic and urbanization factors in all steps of the model, we retained human demographic PC1 and PC2 throughout the model selection procedure but otherwise removed the least significant predictor variable (if individual variable *P*>0.10) if the removal step led to a better fit according to Akaike's information criterion (AIC). This model-fitting procedure let us explore which variables had the greatest power in explaining variation in human incidence of human WNV while also simplifying toward a minimal adequate model. Following this procedure, we constructed two regression models, one that explained 2002 human WNV incidence by 1998 avian community metrics, and a separate model that explained 2002 human WNV by 2002 avian community metrics. We have included a correlation matrix of all the relevant contrasts in avian community structure metrics in Appendix S1 and S2.

We also investigated whether avian community structure metrics changed from 1998 to 2002 with a repeated measures ANOVA with year as a within-subjects variable and county infection status (i.e., reporting human infection or no infection) as a between-subjects.

All statistical analyses were performed with SPSS v15.0 employing two-tailed tests of probability. We log- or square root-transformed variables as appropriate to meet the normality assumptions of parametric statistical tests.

## Results

**Figure 2:**
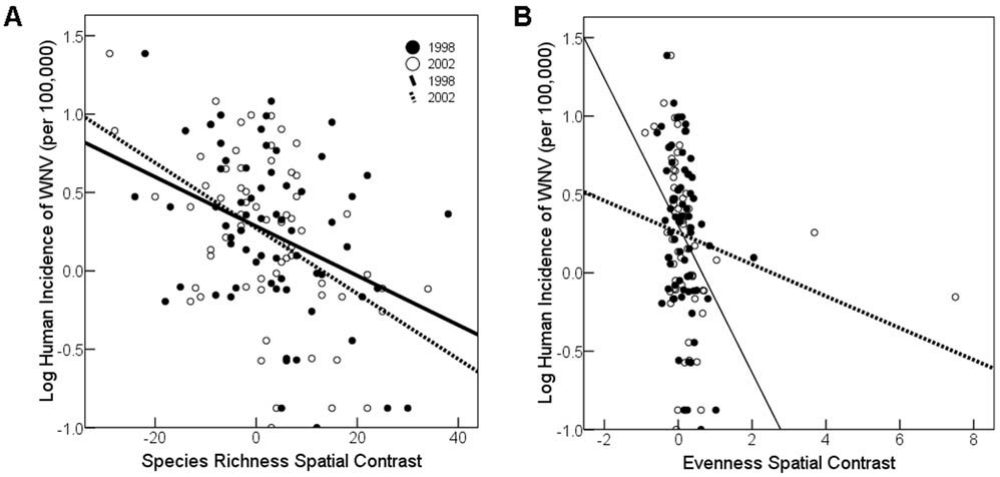
Plots of log human incidence of WNV (per 100,000 people) on (A) species richness contrasts constructed from the difference between neighboring pairs of counties; and (B) Shannon-Weiner evenness contrasts. Filled circles represent data from 1998 and open circles represent reports from 2002. The solid line is best-fit linear regression line for 1998 and the dotted line represents the regression line for 2002.

In both the 1998 and 2002 partial correlation analyses, the spatial contrast of avian species richness was strongly negatively related to the contrast in human incidence of WNV (species richness in 1998 predicted human disease in 2002: *r*_61_ = −0.358, *P* = 0.004; richness in 2002 also predicted disease in 2002: *r*_61_ = −0.509, *P*<0.001; Fig. 2A) and these relationships did not differ between years (ANCOVA, *F*_1,125_ = 1.11, *P* = 0.294). There was also a negative but weaker relationship between the contrast of avian species evenness in 1998 and the contrast in human WNV in 2002 (*r*_61_ = −0.333, *P* = 0.008) and this relationship was somewhat diminished when comparing evenness contrast in 2002 with human WNV contrast in that same year (*r*_61_ = −0.221, *P* = 0.081; Fig. 2B). The change in slope of the evenness-disease relationship from 1998 to 2002 was not quite significant (*F*_1,125_ = 3.52, *P* = 0.063). If the apparent outlier in the 2002 evenness contrast dataset is removed, the relationship between 2002 evenness and disease becomes stronger, more negative, and more similar to the 1998 analysis (*r*_61_ = −0.268, *P* = 0.035). However, there is no biological reason to exclude this datum, hence we have included it in further analyses.

Interestingly, neither measure of total community diversity was eroded from 1998 to 2002 (repeated-measures ANOVA, richness increased non-significantly: *F*_1,128_ = 3.78, *P* = 0.054; evenness: *F*_1,128_ = 1.37, *P* = 0.244), further supporting the notion of a fairly robust diversity-disease relationship even once WNV has impacted avian populations. However, it is clear that a difference in species richness is generally a better predictor of human WNV prevalence than a difference in community evenness.

To explore whether the contrasts of relative or absolute abundance of the select avian families (and American robins) were better predictors of human WNV incidence we performed a series of partial correlations (controlling for human demographic PC1 and PC2) and tested whether the effect sizes (i.e., the unsigned partial correlation coefficients) were larger for relative versus absolute measures of abundance with a Wilcoxon matched-pairs signed rank test, pooling correlations from the 1998 and 2002 analyses together (Table 3). There was no indication that contrasts of absolute measures of abundance were better predictors of human disease than relative measures of abundance (*Z* = 0.764, *N* = 10 pairs, *P* = 0.445).

**Table 3:** Estimates of effect size (unsigned partial correlation coefficients) for the relationships between relative and absolute measures of avian abundance, assessed in 1998 and 2002, with incidence of human WNV in 2002.

Avian taxa	Absolute abundance	Relative abundance
Corvids 1998	0.212	0.184
Old World Sparrows 1998	0.018	0.002
American robins 1998	0.006	0.164
Thrushes 1998	0.031	0.034
Finches 1998	0.110	0.236
Corvids 2002	0.061	0.023
Old World Sparrows 2002	0.092	0.235
American robins 2002	0.034	0.104
Thrushes 2002	0.024	0.013
Finches 2002	0.058	0.053

Our regression model selection procedure generated highly significant linear models relating the spatial contrast of 2002 human disease incidence to 1998 avian community structure contrasts (*F*_8,56_ = 6.53, *r* = 0.695, *r* ^2^ = 0.483, *P*<0.001 , AIC = −111.038) and 2002 spatial contrasts of avian community structure (*F*_6,58_ = 9.89, *r* = 0.711, *r* ^2^ = 0.506, *P*<0.001 , AIC = −117.99). For the 1998 community analysis, there was a higher incidence contrast of human WNV in 2002 in counties that previously (in 1998) had lower total species richness contrasts (*ß* = −0.028, s.e. = 0.005, 95% CI −0.038 to −0.017, *P*<0.001, partial *r* = −0.514), fewer passerines compared with nonpasserines (*ß* = −0.036, s.e. = 0.015, 95% CI −0.067 to −0.006, *P* = 0.021, partial *r* = −0.228), a lower absolute abundance contrast of American robins (*ß* = −0.009, s.e. = 0.003, 95% CI −0.014 to −0.004, *P* = 0.002, partial *r* = −0.320), but a marginally higher proportion of this same species relative to the total community (*ß* = 8.84, s.e. = 3.37, 95% CI 2.09 to 15.59, *P* = 0.011, partial *r* = 0.252), relatively more corvids (*ß* = 4.13, s.e. = 1.71, 95% CI 0.707 to 7.55, *P* = 0.019, partial *r* = 0.232), a greater total abundance of birds of all species (*ß* = 0.001, s.e. = 0.0002, 95% CI 0.00057 to 0.001, *P*<0.0013, partial *r* = 0.477), and a somewhat more urbanized population (*ß* = 0.233, s.e. = 0.171, 95% CI −0.109 to −0.576, *P* = 0.178, partial *r* = 0.131) that does not vary systematically in socioeconomic status (*ß* = 0.021, s.e. = 0.066, 95% CI −0.112 to 0.153, *P* = 0.755, partial *r* = −0.030). This model does not alter qualitatively if both the non-significant socioeconomic and urbanization metrics are removed. Overall, this model is consistent with the simpler partial correlation analyses, indicating that a greater pre-existing (1998) avian species richness (within the uninfected-infected county pair) is associated with a lower incidence of human WNV cases during the epidemic. Additionally and independently of this diversity-disease relationship, communities in 1998 with relatively more birds overall, and relatively more corvids, robins, and nonpasserine species (contrasted to their nearest neighbor) are more likely to report human WNV once the epidemic occurs (in 2002).

For the 2002 community analysis, there was a higher incidence contrast of human WNV in counties with lower total species richness contrast (*ß* = −0.035, s.e. = 0.005, 95% CI −0.046 to −0.025, *P*<0.001, partial *r* = −0.636), a greater nonpasserine community evenness contrast (*ß* = 0.423, s.e. = 0.175, 95% CI 0.072 to 0.773, *P* = 0.019, partial *r* = 0.223), a marginally greater contrast in relative abundance of house sparrows (*ß* = 2.90, s.e. = 1.78, 95% CI −0.666 to 6.48, *P* = 0.109, partial *r* = 0.150), and a greater contrast in absolute abundance of finches (*ß* = 0.007, s.e. = 0.002, 95% CI 0.003 to 0.010, *P*<0.001, partial *r* = 0.349), and a more urbanized human population (*ß* = 0.390, s.e. = 0.161, 95% CI 0.068 to 0.712, *P* = 0.018, partial *r* = 0.224) that does not vary systematically in socioeconomic status (*ß* = 0.031, s.e. = 0.064, 95% CI −0.097 to 0.158, *P* = 0.632, partial *r* = 0.044). Hence, greater species richness still appears strongly associated with fewer cases of human disease but, surprisingly, higher nonpasserine diversity (measured by evenness) may be an indicator of increased disease risk once the disease has taken hold in the avian population. It would also appear that, comparing the difference between neighboring counties, the absolute abundance of finches and, somewhat, the relative abundance of house sparrows are predictors of increased human WNV. In both the 1998 and 2002 analyses, species richness is notably a stronger predictor of human WNV incidence than any measure of community evenness. Also, there was a general trend for urbanization to be positively associated with a greater incidence of disease independently of the community structure-disease correlations.

Although we indicated that overall community evenness does not change within counties from 1998 to 2002, nonpasserine evenness significantly declines (repeated-measures ANOVA, *F*_1,128_ = 11.21, *P* = 0.001) and this pattern does not differ between counties that do or do not report human cases of WNV (*F*_1,128_ = 0.032, *P* = 0.858). However, the opposite pattern is observed among passerines, with evenness increasing from 1998 to 2002 (*F*_1,128_ = 11.25, *P* = 0.001) with no difference in this increase in counties that do or do not report human WNV cases (*F*_1,128_ = 0.475, *P* = 0.492). These differences suggest that fundamentally different population processes are occurring for nonpasserines and passerines over the period that avian WNV becomes established in these 130 counties. We did not detect any changes in the relative or absolute abundance of corvids, house sparrows, or finches from 1998 to 2002 (*F*_1,128_<1.09, *P*>0.299, in all cases). However, there was a slight increase in the relative (*F*_1,128_ = 6.32, *P* = 0.013) and absolute abundance (*F*_1,128_ = 3.26, *P* = 0.073) of thrushes, which was also associated with a large relative increase (*F*_1,128_ = 17.09, *P*<0.001) and absolute increase (*F*_1,128_ = 9.34, *P* = 0.003) in the abundance or American robins. This increase in robins did not differ between counties that did or did not report human cases of WNV (*F*_1,128_ = 0.212, *P* = 0.646).

## Discussion

The geospatial contrast in species richness, either assessed in 1998 or in 2002, was strongly negatively related to the disease prevalence in 2002, which supports our hypothesis that subtle differences in avian diversity between neighboring counties helps buffer humans against WNV infection. Even once WNV is established in all counties and the human epidemic is in full swing (in 2002), the between-county contrast in species richness remains strongly negatively associated with the incidence of human disease. Overall, avian community structure can explain approximately 50% of the variation in human WNV incidence, which seems a high proportion given the rather indirect mechanistic links that most likely underlie these relationships.

Of the possible mechanisms that could help explain a general diversity-disease relationship, our data are not wholly consistent with either transmission reduction or encounter reduction being the major mechanisms (Table 1). For example, under transmission reduction, the encounters between mosquito vectors and infected, highly competent avian hosts can be reduced when relatively more lower-competence hosts are introduced into the population [1]. Therefore, the density and relative abundance of lower-competence hosts is more important than the mere presence/absence of lower-competence hosts in driving this mechanism. If transmission reduction predominated, we would expect to see a stronger negative relationship between human incidence of WNV and community evenness (which incorporates aspects of relative abundance of all host species) than the negative relationship between human WNV and avian species richness (which is an index of presence/absence of host species). This was not the case. We found that the geospatial contrast of species richness was consistently a better predictor of human disease. A similar argument exists for encounter reduction, where encounters between hosts and infected vectors should decrease where there is a greater relative abundance of new species. Therefore, we cannot support transmission reduction or encounter reduction as being the major mechanisms driving the patterns we observed here. However, because our analyses are correlational, it is premature to rule out these mechanisms entirely.

We ruled out the vector regulation mechanism by including a proxy for mosquito density in our analyses (i.e., the urbanization metric) and we do not have data to assess the merits of the recovery augmentation mechanism. Therefore, we are left to consider the density-dependent mechanism of susceptible host regulation. Our analysis of the effect sizes of absolute versus relative abundance contrast metrics in explaining human WNV incidence indicates that these measures have approximately equal explanatory value, which is not entirely consistent with the mechanism of susceptible host regulation. However, the geospatial contrast of total abundance of all avian (i.e., potential host) species in 1998 was a good predictor of human WNV incidence in 2002, with more disease occurring in neighboring counties with a higher density of potential hosts. Contrary to what has been assumed for many vector-borne diseases [1], [31] this pattern is consistent with a density-dependent mechanism of disease transmission, which further erodes confidence in frequency-dependent explanations of how WNV is transmitted from birds to humans.

Overall, we are left with partially supporting the mechanism of susceptible host regulation and down-playing the roles of transmission and encounter reduction in explaining the diversity-disease relationship we show here. We do not intend to overstate these conclusions as our data are not experimental, which obfuscates any statements concerning causality in mechanisms, and we can also not exclude that there are other mechanisms that are yet to be formalized that can explain the general patterns in our data. However, we hope that this form of analysis helps others to consider further the relative strengths of mechanisms that can lead to a diversity-disease relationship.

Unexpectedly, the 2002 community analyses indicate that a greater contrast in community evenness (i.e., relative diversity) of nonpasserines may actually be associated with more cases of human disease, not less. This is consistent with this group of birds contributing transmission and/or encounter augmentation [1], which has not been suggested previously, and runs somewhat contrary to the one previous analysis of avian diversity and human WNV [4]. Therefore, we recommend further testing of nonpasserine species in monitoring WNV as our analyses are consistent with nonpasserine species being more effective viral reservoirs than is currently believed and/or that transmission of the virus among nonpasserine host species is more likely than transmission within a nonpasserine species [1].

The geospatial contrasts of relative abundance of corvids and of American robins before WNV was reported in the US (i.e., in 1998) were both moderate but independent predictors of future human infection. This pattern is somewhat consistent with a recent claim that American robins are largely responsible for transmission rates from birds to humans, because of mosquito vectors' affinity for feeding from robins [10], but also reiterates the likely role that highly disease-susceptible corvids can play in determining public health risks [27]. The role of the American robin in WNV epidemics is further implicated in our data by the increase in relative and absolute abundance of this species from 1998 to 2002. Although this increase in robins did not differ between counties that did or did not report human WNV, this pattern is still consistent with American robins being positively associated with the overall (human and avian) WNV epidemics. The 2002 community analysis also indicated that the geospatial contrast of absolute abundance of finches is positively associated with the contrast in human WNV incidence. This is not associated with an increase in finch abundance from 1998 to 2002 and suggests that this avian family may play an important role in determining a human epidemic [24].

We do not intend to put too much stock in interpreting the changes in particular avian families over the time period we studied as these data are correlational and many other factors could have changed in these counties from 1998 to 2002. However, we were surprised to see a lack of change in abundance of several passerine groups that are known to be affected by WNV. In particular, we were expecting to see a noticeable decrease in corvids [15], [22] and thrushes [15], but observed neither pattern. Similarly, we were expecting to see a decrease in total avian diversity metrics associated with WNV, but did not observe this pattern. Both sets of (lack of) patterns may indicate that avian populations, at least those surveyed by BBS methods, are surprisingly resilient during the first years of a disease epidemic [12].

Surprisingly, we recorded an increase in passerine community evenness from 1998 to 2002, further indicating that many more passerine species than is commonly accepted could be resilient to WNV. There is a fairly widespread perception that nonpasserines are, as a group, more resistant to WNV infection than passerines [10], [15], [16]. However, if this were the case, we would expect to see a greater loss of passerine than nonpasserine diversity during this period. As we observed the opposite, we hypothesize that there are many passerines that have not been studied in detail that may be poor viral hosts and, perhaps, many nonpasserine species that are more effective viral replicators than currently believed. Therefore, although substantial efforts have been made to compare the viral host properties of several species [16], [18], [24], we propose there is a need for yet broader taxonomic testing among birds.

Other than associations between our geospatial contrasts of avian community structure and human disease, WNV incidence was also weakly predicted by our index of urbanization. This pattern could exist for many, non-mutually exclusive reasons [32]. For example, as people live at higher density in urbanized counties there may be greater contact rates among people and between people and wildlife. Wildlife habitat fragments are likely to be smaller in urbanized landscapes, also making contact rates among wildlife higher in such areas. Additionally, the major mosquito vectors in the eastern US are urban-associated [21], [29], [30], often breeding in small ephemeral pools that occur in poorly drained urban areas. We may expect WNV infection risk to be higher in such areas. Hence, it is perhaps not surprising that more urbanized counties are more likely to report human cases of WNV. However, independently of this association between urbanization and human disease, fairly small differences in avian community diversity, as assessed by the difference in diversity between neighboring counties, explained a surprisingly large amount (approximately 50%) of the between-county difference in human incidence of WNV. These contrast differences could be used by local and state health authorities to interpret how alterations to standing avian community structure can alter the relative occurrence of human disease in particular areas. Therefore, our analyses provide support for the growing view that wildlife diversity can help buffer human populations from infectious diseases [review in 1] when such diseases, which can emerge by natural or anthropogenic means, replicate within wildlife and spill over into the human population. Therefore, we hope to stimulate further consideration of avian (or any disease host) community structure in public health and safety strategies and point to the increasing evidence for economically valuable ecosystem services provided by biodiversity.

## Supporting Information

Appendix S1 Correlations of 1998 community structure contrasts
(0.04 MB PDF)

Appendix S2 Correlations of 2002 community structure contrasts
(0.04 MB PDF)
