# Supplementary material for: Increased Avian Diversity Is Associated with Lower Incidence of Human West Nile Infection: Observation of the Dilution Effect
Source: PLoS One. 2008 Jun 25;3(6):e2488. doi: 10.1371/journal.pone.0002488 (PMC2427181; doi:10.1371/journal.pone.0002488)
Supplement: Appendix S1 — Correlations of 1998 community structure contrasts (0.04 MB PDF) [file pone.0002488.s001.pdf]

Appendix S1. Pearson correlations (*r*) among contrast metrics (i.e., difference in metric between neighboring counties that did or did not report human cases of WNV) for the 1998 linear model analyses. *P* values are two-tailed and have not been adjusted for the 153 comparisons reported here, so should be interpreted cautiously and with an appropriately adjusted alpha value. *N* = 65 for all cases.

		Species richness	Total evenness	Human PC1	Human PC2	Prop passerines: nonpasserines	Prop of Corvidae	Prop of Passeridae	Prop of American robins	Prop of Turdidae	Prop of Fringillidae	Nonpasserine evenness	Passerine evenness	Number of Corvidae	Number of Passeridae	Number of American robins	Number of Turdidae	Number of Fringillidae
Total evenness	*r*	0.489																
	P	< 0.001																
Human PC1	*r*	-0.095	-0.034															
	P	0.449	0.790															
Human PC2	*r*	-0.045	-0.161	-0.192														
	P	0.724	0.200	0.126														
Prop passerines:nonpasserines	*r*	-0.176	-0.019	-0.029	-0.078													
	P	0.161	0.882	0.820	0.538													
Prop of Corvidae	*r*	-0.029	0.392	0.076	-0.207	-0.060												
	P	0.819	0.001	0.550	0.098	0.636												
Prop of Passeridae	*r*	0.093	-0.030	0.131	-0.069	0.199	-0.056											
	P	0.462	0.814	0.297	0.584	0.113	0.658											
Prop of American robins	*r*	-0.163	-0.235	0.047	0.039	0.066	-0.081	0.058										
	P	0.195	0.060	0.709	0.761	0.602	0.519	0.647										
Prop of Turdidae	*r*	-0.031	-0.061	-0.052	-0.030	0.231	-0.098	0.022	0.813									
	P	0.809	0.628	0.681	0.810	0.064	0.437	0.863	<0.001									
Prop of Fringillidae	*r*	0.202	0.248	-0.245	0.177	0.070	-0.003	-0.144	-0.285	-0.179								
	P	0.107	0.047	0.049	0.159	0.578	0.983	0.251	0.022	0.153								
Nonpasserine evenness	*r*	0.270	0.222	-0.064	0.056	-0.765	0.044	-0.294	-0.245	-0.306	0.057							
	P	0.030	0.075	0.612	0.655	<0.001	0.725	0.017	0.050	0.013	0.651							
Passerine evenness	*r*	0.316	0.833	0.004	-0.187	0.417	0.351	0.139	-0.086	0.115	0.205	-0.355						
	P	0.010	<0.001	0.974	0.137	0.001	0.004	0.271	0.496	0.362	0.102	0.004						
Number of Corvidae	*r*	0.344	0.100	0.001	-0.004	-0.178	0.520	0.206	-0.084	-0.157	-0.126	0.030	0.078					
	P	0.005	0.429	0.998	0.972	0.157	<0.001	0.100	0.508	0.211	0.316	0.810	0.535					
Number of Passeridae	*r*	0.191	-0.005	0.126	-0.070	0.214	-0.092	0.910	0.026	0.004	-0.155	-0.326	0.180	0.257				
	P	0.128	0.966	0.316	0.582	0.087	0.465	<0.001	0.836	0.978	0.218	0.008	0.151	0.038				
Number of American robins	*r*	0.178	-0.226	0.069	0.050	-0.045	-0.292	0.255	0.681	0.531	-0.270	-0.252	-0.073	0.115	0.237			
	P	0.156	0.071	0.587	0.692	0.723	0.018	0.040	<0.001	<0.001	0.030	0.043	0.563	0.361	0.057			
Number of Turdidae	*r*	0.311	-0.150	0.027	0.019	0.003	-0.286	0.211	0.531	0.576	-0.202	-0.259	0.003	0.231	0.231	0.897		
	P	0.012	0.232	0.833	0.882	0.979	0.021	0.092	<0.001	<0.001	0.106	0.037	0.980	0.064	0.064	<0.001		
Number of Fringillidae	*r*	0.520	0.206	-0.251	0.205	-0.046	-0.142	0.103	-0.269	-0.231	0.769	0.115	0.132	0.205	0.193	-0.054	0.048	
	P	<0.001	0.100	0.044	0.101	0.714	0.258	0.413	0.031	0.064	<0.001	0.360	0.296	0.101	0.123	0.670	0.705	
Total number of birds	*r*	0.499	-0.185	-0.012	0.102	-0.081	-0.337	0.322	0.198	0.145	-0.114	-0.123	-0.107	0.435	0.421	0.711	0.805	0.353
	P	<0.001	0.140	0.923	0.419	0.519	0.006	0.009	0.115	0.248	0.367	0.328	0.395	<0.001	<0.001	<0.001	<0.001	0.004
