# Supplementary material for: Increased Avian Diversity Is Associated with Lower Incidence of Human West Nile Infection: Observation of the Dilution Effect
Source: PLoS One. 2008 Jun 25;3(6):e2488. doi: 10.1371/journal.pone.0002488 (PMC2427181; doi:10.1371/journal.pone.0002488)
Supplement: Appendix S2 — Correlations of 2002 community structure contrasts (0.04 MB PDF) [file pone.0002488.s002.pdf]

Appendix S2. Pearson correlations (*r*) among contrast metrics (i.e., difference in metric between neighboring counties that did or did not report human cases of WNV) for the 2002 linear model analyses. *P* values are two-tailed and have not been adjusted for the 153 comparisons reported here, so should be interpreted cautiously and with an appropriately adjusted alpha value. *N* = 65 for all cases.

		Species richness	Total evenness	Human PC1	Human PC2	Prop passerines: nonpasserines	Prop of Corvidae	Prop of Passeridae	Prop of American robins	Prop of Turdidae	Prop of Fringillidae	Nonpasserine evenness	Passerine evenness	Number of Corvidae	Number of Passeridae	Number of American robins	Number of Turdidae	Number of Fringillidae
Total evenness	*r*	0.340																
	P	0.006																
Human PC1	*r*	0.014	-0.052															
	P	0.909	0.683															
Human PC2	*r*	0.099	0.054	-0.192														
	P	0.433	0.672	0.126														
Prop passerines:nonpasserines	*r*	-0.159	-0.167	0.067	-0.092													
	P	0.206	0.184	0.598	0.467													
Prop of Corvidae	*r*	-0.043	0.073	-0.036	-0.099	0.009												
	P	0.734	0.562	0.774	0.431	0.940												
Prop of Passeridae	*r*	0.077	-0.118	0.137	0.041	-0.050	-0.114											
	P	0.544	0.349	0.277	0.745	0.695	0.368											
Prop of American robins	*r*	-0.273	0.144	-0.086	-0.008	0.099	0.079	0.103										
	P	0.028	0.253	0.495	0.948	0.434	0.530	0.415										
Prop of Turdidae	*r*	-0.200	0.107	-0.018	-0.122	0.147	0.157	0.013	0.903									
	P	0.110	0.398	0.889	0.332	0.241	0.212	0.920	<0.001									
Prop of Fringillidae	*r*	0.366	0.023	0.338	-0.051	0.228	0.122	0.048	-0.195	-0.110								
	P	0.003	0.854	0.006	0.688	0.068	0.331	0.707	0.120	0.384								
Nonpasserine evenness	*r*	0.218	0.712	-0.111	0.156	-0.681	-0.079	-0.097	0.001	-0.077	-0.188							
	P	0.081	<0.001	0.380	0.213	<0.001	0.533	0.443	0.995	0.542	0.134							
Passerine evenness	*r*	0.338	0.972	-0.025	0.012	0.027	0.115	-0.110	0.174	0.155	0.091	0.527						
	P	0.006	<0.001	0.841	0.922	0.834	0.361	0.382	0.166	0.218	0.470	<0.001						
Number of Corvidae	*r*	0.199	-0.131	0.007	-0.029	0.024	0.658	0.206	0.056	0.093	0.214	-0.223	-0.084					
	P	0.113	0.298	0.955	0.816	0.851	<0.001	0.100	0.657	0.459	0.087	0.074	0.508					
Number of Passeridae	*r*	0.209	-0.071	0.096	0.007	-0.003	-0.188	0.795	0.068	0.036	0.006	-0.113	-0.048	0.160				
	P	0.095	0.574	0.449	0.957	0.979	0.133	<0.001	0.590	0.774	0.963	0.370	0.704	0.202				
Number of American robins	*r*	0.059	-0.001	0.010	0.051	-0.018	-0.142	0.350	0.705	0.612	-0.129	-0.038	0.012	0.232	0.408			
	P	0.642	0.996	0.940	0.689	0.886	0.259	0.004	<0.001	<0.001	0.304	0.764	0.924	0.063	0.001			
Number of Turdidae	*r*	0.151	-0.031	0.038	0.011	-0.005	-0.125	0.348	0.624	0.646	-0.093	-0.084	-0.009	0.287	0.467	0.951		
	P	0.230	0.808	0.764	0.933	0.969	0.322	0.004	<0.001	<0.001	0.462	0.504	0.944	0.021	<0.001	<0.001		
Number of Fringillidae	*r*	0.526	-0.041	0.141	0.025	0.061	-0.073	0.340	-0.139	-0.109	0.685	-0.121	-0.009	0.413	0.470	0.274	0.337	
	P	<0.001	0.747	0.263	0.845	0.628	0.562	0.006	0.268	0.387	<0.001	0.338	0.944	0.001	<0.001	0.027	0.006	
Total number of birds	*r*	0.441	-0.121	0.011	0.098	-0.074	-0.290	0.431	-0.053	-0.051	0.033	-0.086	-0.117	0.299	0.703	0.537	0.619	0.653
	P	<0.001	0.339	0.931	0.439	0.555	0.019	<0.001	0.675	0.687	0.795	0.497	0.353	.015	<0.001	<0.001	<0.001	<0.001
